# Severe experimental folate deficiency in a human subject - a longitudinal investigation of red-cell folate immunoassay errors as megaloblastic anaemia develops

**DOI:** 10.1186/2193-1801-3-441

**Published:** 2014-09-23

**Authors:** Paul Henry Golding

**Affiliations:** Unit 5, 18 Webster Road, Nambour, QLD 4560 Australia

**Keywords:** Red-cell folate immunoassays, Immunoassay errors, Megaloblastic anaemia, Diagnosis of folate deficiency

## Abstract

**Background:**

The few published studies comparing results between commercial red-cell folate immunoassays have found significant differences. None have provided longitudinal data during the development of megaloblastic anaemia from severe folate deficiency. The objective was to produce longitudinal data, comparing results between three commercial immunoassays for red-cell folate, generated by means of severe experimental folate deficiency.

**Methods:**

This 58 year old male, initially replete in folate, used a folate-deficient diet to severely deplete himself of folate until overt megaloblastic anaemia developed. The Siemens Advia Centaur, Roche Elecsys 2010 and Beckman UniCel DxI 800 Access immunoassay systems were used, by different clinical pathology laboratories, to perform weekly assays for red-cell folate throughout the depletion stage. The results were analysed graphically four ways: comparison with lines of equality; number of standard deviations difference against the means; number of standard deviations difference over time; variation over time.

**Results:**

There were very significant differences, varying with time and folate concentration, between the results reported by the three laboratories. The differences were greatest, up to 17 standard deviations, between the Siemens Advia Centaur and each of the other two systems. Of the 85 results comparing the Siemens Advia Centaur and the Roche Elecsys 2010, two were within the 99.9% confidence interval. Of the 91 results comparing the Siemens Advia Centaur and the Beckman UniCel DxI 800 Access, 22 were within the 99.9% confidence interval. Of the 83 results comparing the Beckman UniCel DxI 800 Access and the Roche Elecsys 2010, 37 were within the 99.9% confidence interval.

**Conclusions:**

Comparative longitudinal data from clinical pathology laboratories, produced during experimental folate deficiency, have exposed very significant differences in results between commercial red-cell folate immunoassays. One immunoassay, the Roche Elecsys 2010, failed to detect overt megaloblastic anaemia of severe folate deficiency.

**Electronic supplementary material:**

The online version of this article (doi:10.1186/2193-1801-3-441) contains supplementary material, which is available to authorized users.

## Background

Folate deficiency increases the risk of anaemia, neural tube defects, cancer, vascular disease and neurological disorders (Bailey 1995, 2009). In addition to dietary inadequacy, a functional folate deficiency can be caused by anything that interferes with the bioavailability of the folate, including any defect in the hydrolysis, absorption, transport or cellular utilization (McNulty and Pentieva 2009; Gregory 1995). Folate antagonists include alcohol, and medicines used for the treatment of cancer, rheumatoid arthritis, inflammatory bowel disease and other disorders (McNulty and Pentieva 2009; Gregory 1995). Gastro-intestinal disease or mutations in genes for folate transport or metabolism also affect folate bioavailability (McNulty and Pentieva 2009). A deficiency of vitamin B_12_ can cause a secondary, functional, folate deficiency by means of the methylfolate trap (Tisman and Herbert 1973; Smulders et al. 2006; Scott and Weir 1981).

Because a folate-replete diet cannot guarantee adequate folate bioavailability, even where there is mandatory food folate fortification, there remains a need for reliable clinical pathology tests for folate. Microbiological assays and mass spectrometry methods are not routinely used outside of the research environment. Because of their lower cost and higher throughput, commercial automated immunoassays are used for routine patient testing (Pfeiffer et al. 2009).

Published studies have reported significant differences in folate results between methods (Nakazato et al. 2012; Clifford et al. 2005; Gunter et al. 1996; Jacobsen 1996; Callen 2010; Owen and Roberts 2003). There have been calls for the standardization of reference intervals for red-cell folate, to allow results to be compared between laboratories and to establish minimum safe concentrations. In reporting the results of an international method comparison study, Gunter (1996) concluded: *“The overall results underscore the urgent need for developing and validating reference methods for serum and whole-blood folate and for properly characterized reference materials”*. In a related editorial, Jacobsen (1996) stated: *“A task force should be established and given the charge of troubleshooting folate assay problems and establishing reference methods”*. Wright et al. (1998) stated: “*Assessment of the assumptions that underpin RCF assays indicates that many are false*”.

Despite these calls for change, the problems with the assay of red-cell folate, in a clinical setting, remain. Clifford (2005) reported: “*We conclude that RBC folate levels are assay dependent, as is the definition of optimized status; there continues to be a need for an accurate assay of RBC folate*”. Wickramasinghe (2006) stated: “*The accuracy of folate assays and particularly of fully-automated red cell folate assays is questionable.*” According to Pfeiffer et al. (2009): *“much more needs to be done to achieve better comparability between methods”*.

Two published studies compared the three commercial automated red-cell folate immunoassay systems used in this experiment; Siemens Advia Centaur, Roche Elecsys 2010 and Beckman UniCel DxI 800 Access (Callen 2010; Owen and Roberts 2003). According to Callen, these three systems account for 61% of instruments used for assay of red-cell folate in Australia. Both studies reported significant differences between results for the three systems. There is no published longitudinal data comparing immunoassay methods, in a clinical setting, during the development of severe folate deficiency.

In 2009, while performing a self-experiment to investigate tests for vitamin B_12_ deficiency, this author monitored his folate concentrations to check for the possibility of secondary, functional, folate deficiency caused by the methylfolate trap (Tisman and Herbert 1973; Smulders et al. 2006; Scott and Weir 1981).

The three red-cell folate immunoassay systems produced very significantly different results, (unpublished data, available from this author). Repeated testing, with samples collected within a two-hour window, produced results from one system that were three times the red-cell folate concentration reported by another. Because these three systems account for 61% of instruments used for assay of red-cell folate in Australia (Callen 2010), and one system alone accounts for 33%, such errors could affect many patients.

Because pregnancy increases the demand for folate, and because of the risk of neural-tube defects in children of folate-deficient mothers, significant errors in the measurement of red-cell folate could have very serious consequences. Other patients at high risk of folate deficiency, including those taking specific medicines or with gastro-intestinal disease or certain genetic disorders, could also suffer serious harm.

Commencing in May 2011, this author used himself as the subject of an experiment to investigate the discrepancies between the three red-cell folate immunoassays, the subject of this report. The biochemical and haematological responses to the resulting severe folate deficiency have been described in detail in a separate report (Golding 2014).

### Objectives

The objective of this investigation was to produce longitudinal data, comparing results between three commercial immunoassays for red-cell folate, generated by means of severe experimental folate deficiency.

## Methods

### Ethics statement

As a member of COPE (Committee on Publication Ethics), SpringerPlus requires that experiments on human subjects adhere to the ethical standards of the Declaration of Helsinki. In particular, there must be informed consent of subjects, and the experiment must be approved and overseen by a research ethics committee or institutional review board. Because this author did not obtain informed consent, and the study did not receive ethics committee approval, it is necessary for the author to explain the reasons why publication of this report is ethical.

Firstly, the author was both the experimenter and the single subject, so the requirement for informed consent does not apply. There was no institutional involvement, so there was no possibility of coercion. The subject was assessed by a psychiatrist before the experiment commenced, and found to be competent to evaluate the risks and benefits, and to accept full responsibility for the conduct of the experiment.

Secondly, the Declaration of Helsinki is silent on self-experimentation, because it is concerned with the conduct of research on patients or healthy volunteers by others. The requirement for ethics committee approval therefore does not apply where the single subject is also the sole experimenter. Also, because there was no institution involved in the study, with the experiment conducted by an independent researcher, no ethics committee existed.

Thirdly, the experiment was not performed recklessly or carelessly; the subject’s condition was monitored weekly by the psychiatrist. This doctor is a Fellow of the Royal Australian and New Zealand College of Psychiatrists, and had no conflict of interests. Being a qualified medical practitioner receiving all weekly pathology reports, he was able to continually assess the condition of the subject. For safety, it was agreed at the outset that he would take control if, but only if, there was an immediate life-threatening condition. The subject instigated, designed and performed the experiment, and this doctor’s only role was monitoring for safety.

Lastly, the motivation for performing the experiment was ethical, and involved no conflict of interests. The author wanted to investigate the gross differences between the red-cell folate immunoassays, as found during his experiment of 2009, because he was aware of the potential consequences of errors in measurement of folate concentrations, especially in pregnant women and others at high risk of deficiency. The author was motivated only by the desire to gain and share knowledge, to advance medical science, for the benefit of patients.

### Experiment design

A human subject, initially replete in folate, consumed a folate-deficient diet to severely deplete the body of folate, culminating in megaloblastic anaemia. Precautions were taken to avoid the confounding effects of other nutrient deficiencies. The biochemical and haematological responses were monitored by weekly or twice-weekly blood tests during both the depletion and recovery stages. Three commercial clinical pathology laboratories, all accredited by the Australian National Association of Testing Authorities (NATA), were used to increase confidence in the results. One research laboratory, specializing in microbiological folate assays, was used to confirm severe folate depletion of the subject. Megaloblastic anaemia was confirmed by blood cell counts, blood films, and bone marrow examination.

### The subject

The subject was this author, a 58 year old male non-drinker, with no history of folate deficiency or anaemia. He had for many years consumed a vegetarian diet replete in folate, and had never taken folate supplements. There are no reported interferences, with any of the analytes monitored, from any medication taken by the subject. Because of a history of vitamin B_12_ deficiency, of uncertain cause, the subject had been taking 1000 μg oral methylcobalamin daily for two years immediately prior to this study. Extensive testing showed consistently normal results for serum vitamin B_12_ and the two metabolites, homocysteine and methylmalonic acid, at this level of intake.

### The folate-deficient diet

The folate-deficient diet was a modified vegetarian version of that used by Herbert (1962, 1963). Several variations were made during the course of the experiment to improve the balance between folate reduction and energy maintenance. This was necessary to minimize the significant weight loss caused by the semi-starvation diet. The average daily dietary folate intake, during the depletion stage, varied from 25 μg to 2 μg, or 6% to 0.5% of the recommended daily intake (RDI) of 400 μg (Institute of Medicine, National Academy of Sciences 1998). The folate concentration for each food item was taken from the data provided by Food Standards Australia and New Zealand (2011). For the last ten months of folate starvation, the diet consisted primarily of white rice, boiled and washed three times, flavoured with either salt or coconut and sugar; additional energy was supplied by Gatorade and barley sugar. Compliance with the diet was 100%.

### Blood sampling

#### Blood sample collection

Precautions were taken to ensure that consistent and valid blood samples were received by the laboratories. Each of the three laboratories that performed the routine biochemistry and haematology tests collected their own samples, using professional phlebotomists at government-approved collection centres. In the morning of each sampling day, the blood samples were all collected within the narrowest possible time window; usually not more than two hours from first to last. The subject always fasted overnight, and was well hydrated, ensuring maximum possible consistency between samples. The phlebotomy technique use by each collector was chosen to provide the highest quality samples; tourniquet application was carefully controlled, and discard tubes were used where required. All samples were promptly transported from the collection centre to the laboratory, cooled on ice, to avoid deterioration.

#### Blood sampling frequency

The frequency of routine blood sampling was adapted according to the rate of change of the biochemical and haematological responses. Blood samples were initially collected weekly; this was increased to twice weekly for the last three months of the folate depletion stage and the first month of the recovery stage.

### Folate immunoassays

#### Immunoassay red-cell folate

All three commercial clinical pathology laboratories were used to assay red-cell folate, using automated immunoassay systems. Laboratory A used the Siemens Advia Centaur; Laboratory B used the Roche Elecsys 2010; Laboratory C used the Beckman UniCel DxI 800.

There was a change in method for the Roche Elecsys 2010, towards the end of the folate-depletion stage (Roche Diagnostics 2011). The change was only in the instrument software; the hardware and reagents remained unchanged. This modification, to the internal analyser software, significantly reduced the sensitivity of the instrument. Several later results were therefore reported only as *“< x nmol/L*”, rather than as a numerical result, even for red-cell folate concentrations apparently greater than 1000 nmol/L.

#### Immunoassay serum folate

Laboratories A and B reported serum folate using the same systems used for red-cell folate; Laboratory C did not report serum folate.

### Folate microbiological assays

Microbiological serum and red-cell folate assays, of a single blood sample collected on day 574, were used to confirm severe folate depletion of the subject. The assays were performed by the Food Science and Human Nutrition Department, University of Florida. The procedure, based on that described by O’Broin and Kelleher (1992), was developed by the Centers for Disease Control and Prevention (Zhang et al. 2008).

Special precautions were taken, during collection and transport of the blood samples, to ensure that valid samples were received by the laboratory. Serum samples were collected in serum separator tubes, and whole blood samples were collected in EDTA tubes. The serum tubes were spun down at the collection centre, and all samples were immediately transported, cooled on ice, to the local laboratory. Aliquots of serum and whole blood were then transferred to cryovials and frozen to -80°C. The samples were sent, packed in dry ice, to the laboratory in Florida by specialized international courier. All samples were received frozen, and in excellent condition, then stored at -80°C until assayed.

### Homocysteine

Serum total homocysteine was routinely assayed by Laboratory A only, using the Siemens Advia Centaur immunoassay system.

### Haemoglobin

Three clinical pathology laboratories, each equipped with commercially available automated cell counters, were used to monitor the haematological responses to folate depletion. Laboratories A and C used the Sysmex XE-2100 Automated Haematology System; Laboratory B used the Sysmex XS-1000i haematology analyser.

### Bone marrow examinations

Two bone marrow biopsies were performed by an experienced physician, and samples were forwarded to Laboratory C for examination; the first on day 402; the second on day 575. Whole blood, bone marrow aspirate and trephine samples were collected. Marrow aspirate was examined for cellularity, myelogram was performed, and surface markers analysed. Marrow trephine was examined for normality of bone trabeculae, cellularity and infiltration.

### Red-cell folate immunoassay errors - statistical analysis

The differences in results between the three immunoassay methods were analysed using four graphical techniques: comparison of results with lines of equality, including confidence intervals, on an X-Y plot; difference between results, in standard deviations, against the result means; difference between results, in standard deviations, over time; variation in results, with confidence intervals, over time.

#### Comparison with lines of equality

This traditional technique, for comparing results between methods, provides some information but is not considered to be as useful as the difference plot (Bland and Altman 1986; Petersen et al. 1997; Westgard and Hunt 1973). The red-cell folate results from one immunoassay system are plotted on the X axis, and results from another are plotted on the Y axis, for each pair of results. The results, from the two assay methods, may be compared to confidence intervals around the line of equality. Because the confidence intervals are derived from the results on the X axis, with the method used to generate those results effectively treated as a reference method, the magnitude of errors can be overstated. The correlation coefficient may be calculated, but a high correlation does not imply agreement between results from two immunoassay methods (Bland and Altman 1986).

#### Errors against means

This is the technique, for comparing differences in results from two methods, recommended by Bland and Altman (1986). The difference within pairs of red-cell immunoassay results, in standard deviations, is plotted against the mean of each pair of results. This provides information about how the difference in results for two methods is dependent on the mean of the red-cell folate concentration. Because the confidence intervals are derived from the means of the results, the magnitude of errors is less likely to be overstated.

#### Errors over time

This a variation of the technique recommended by Bland and Altman (1986). The difference within pairs of red-cell immunoassay results, in standard deviations, is plotted against time. This provides information about how the difference in results for two methods is dependent on time.

#### Variation over time

The red-cell results from all three immunoassay systems are plotted, with the 95% confidence interval for each method, against time on the same chart. This allows a comparison of all three methods together, showing how the differences between them depend on time.

#### Calculation of errors

The percentage error between results for two different methods is calculated as:


The error, in standard deviations (SD), between two results is calculated as:


Where CVa = coefficient of analytical variation

According to RCPA (2013) and Ricos et al. (2012), for desirable performance, CVa is calculated as:


Where CVi = within-subject coefficient of biological variation

The error, in standard deviations, between two results was therefore calculated as


Where CVi = 12%

The value of CVi for red-cell folate was obtained from Ricos et al. (2012).

## Results

### Data availability

**Figure 1:**
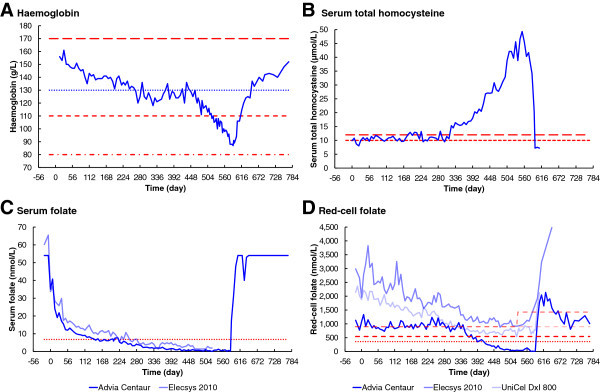
**Changes in analytes over time. A**. Haemoglobin. **B**. Serum total homocysteine. **C**. Serum folate. **D**. Red-cell folate. Severe dietary folate deficiency commenced on Day 0 (23 May 2011). The folate-replete diet, with folate supplementation, resumed on day 586 (29 December 2012), and recovery was complete on day 772 (3 July 2013). The dotted lines in panels **A**, **C** and **D** are the minimum normals, and the long-dashed line in panel **A** is the maximum normal, according to Bates and Lewis (2012). The dotted line in panel **A** is also the WHO (2011) haemoglobin concentration for mild anaemia; the short-dashed line is the WHO concentration for moderate anaemia and the dot-dashed line is the WHO concentration for severe anaemia. The short-dashed line in panel **B** is the upper limit for patients with high coronary risk factors (Stanger et al. 2004), and the long-dashed line is the upper limit in case of normal coronary risk factors (Brouwer et al. 1998). The short-dashed lines in panel **D** are the low end of the reference range, or a minimum cut-off value, as quoted by each testing laboratory.

**Figure 2:**
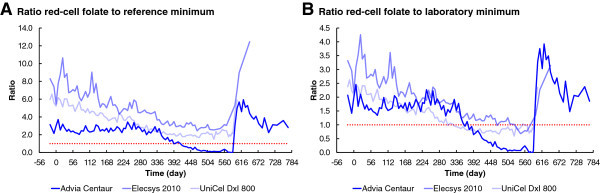
**Ratio of reported red-cell folate to lower limit over time. A**. Ratio red-cell folate to reference minimum. **B**. Ratio red-cell folate to laboratory minimum. The reference minimum, dotted line, in panel **A** is the red-cell folate normal lower limit according to Bates and Lewis (2012). The laboratory minimum, dotted line, in panel **B** is the low end of the reference range, or a minimum cut-off value, quoted by each laboratory.

**Figure 3:**
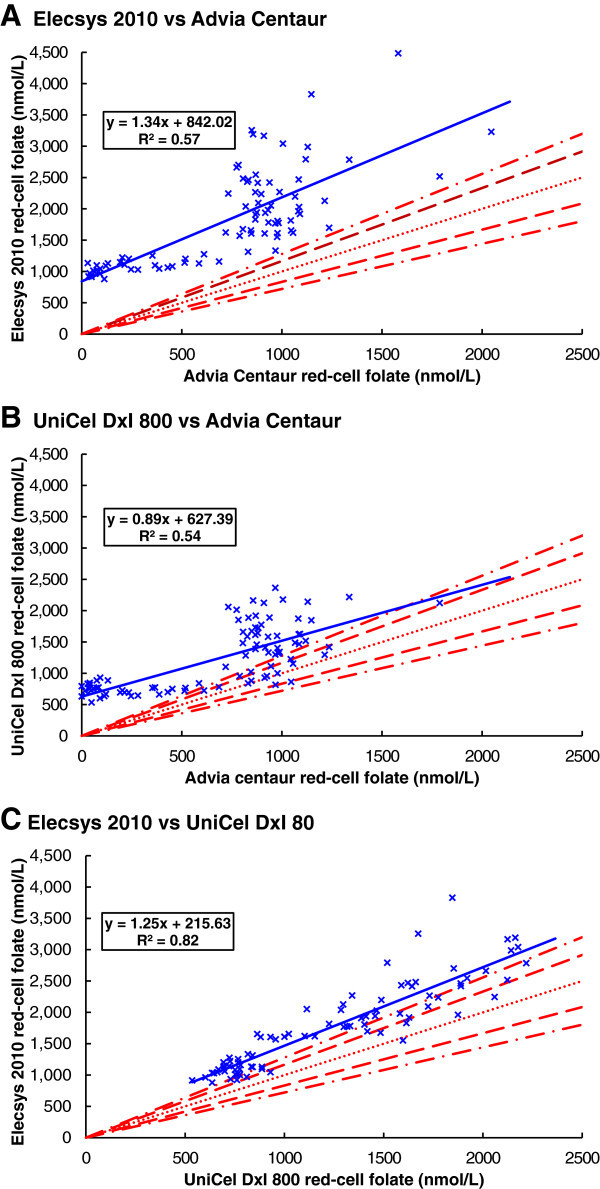
**Comparison of red-cell folate immunoassay results. A**. Elecsys 2010 vs Advia Centaur. **B**. Unicel Dxl 800 vs Advia Centaur. **C**. Elecsys 2010 vs Unicel Dxl 800. The dotted lines are for equality, or zero error. The dashed lines are the 95% confidence interval, and the dot-dashed lines are the 99.9% confidence interval. The solid lines are the linear regression lines.

**Figure 4:**
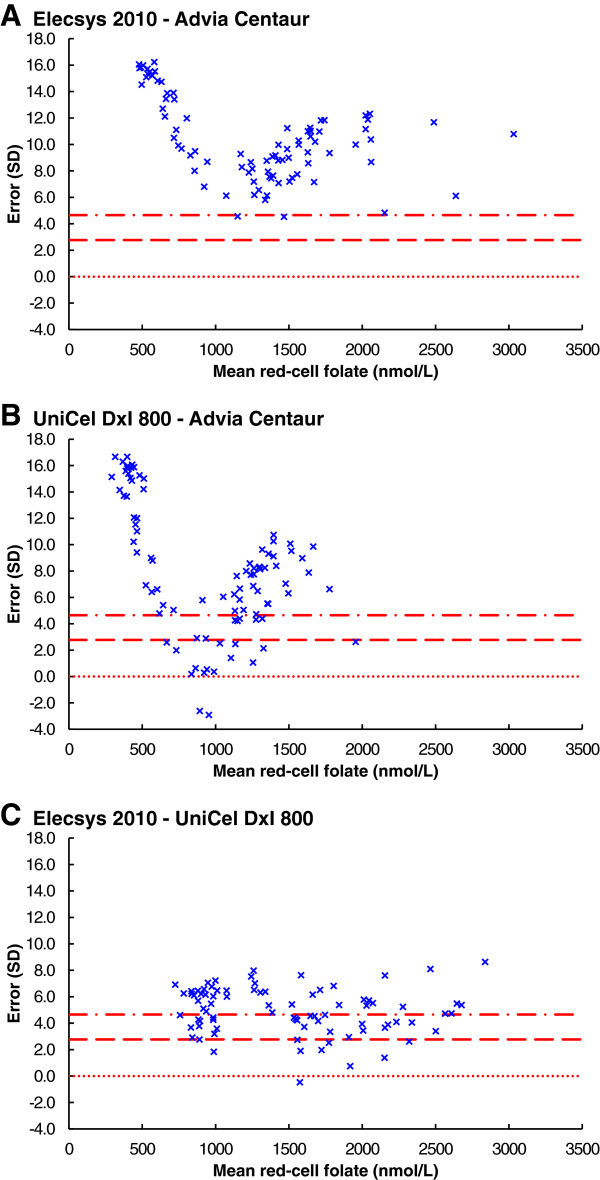
**Red-cell folate immunoassay errors against mean. A**. Elecsys 2010 - Advia Centaur. **B**. Unicel Dxl 800 - Advia Centaur. **C**. Elecsys 2010 - Unicel Dxl 800. The dotted lines are for equality, or zero error. The dashed lines are the 95% confidence interval, and the dot-dashed lines are the 99.9% confidence interval.

**Figure 5:**
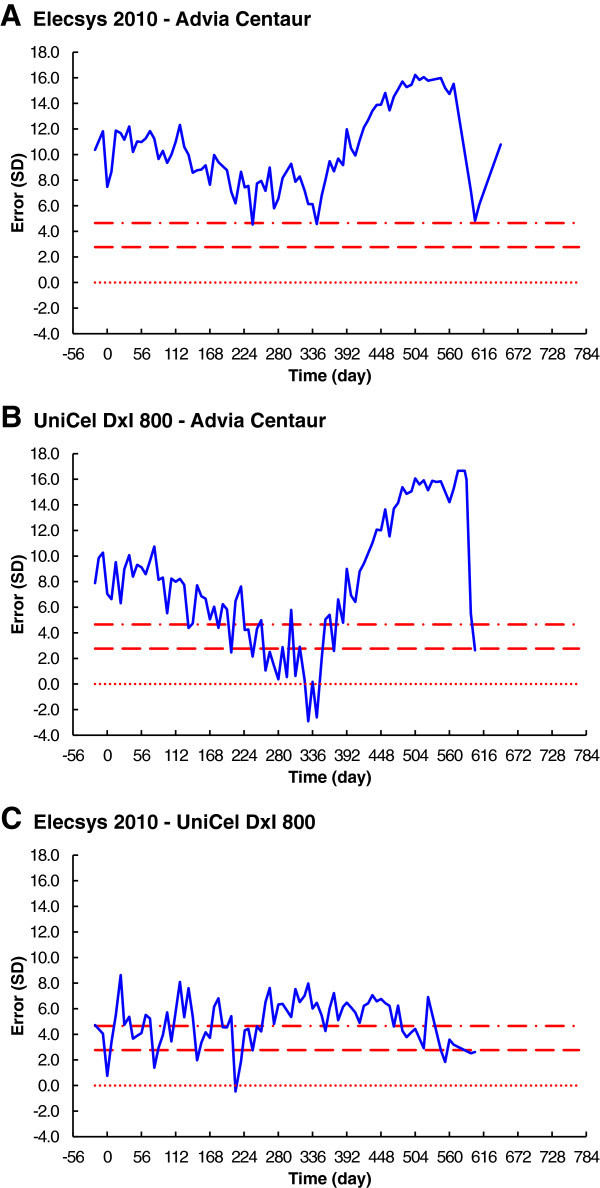
**Red-cell folate immunoassay errors over time. A**. Elecsys 2010 - Advia Centaur. **B**. Unicel Dxl 800 - Advia Centaur. **C**. Elecsys 2010 - Unicel Dxl 800. The dotted lines are for equality, or zero error. The dashed lines are the 95% confidence interval, and the dot-dashed lines are the 99.9% confidence interval.

**Figure 6:**
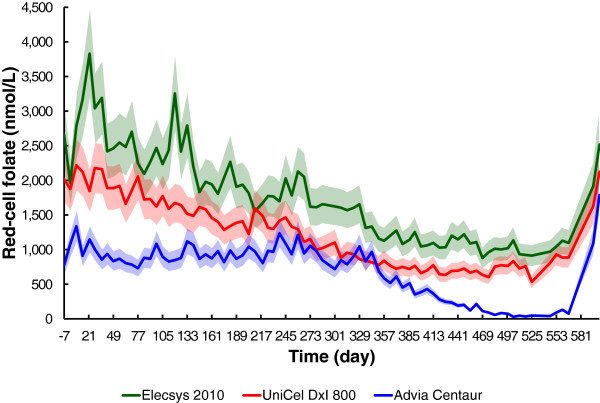
**Red-cell folate and error limits over time.** The solid lines are the reported results. The shaded areas are the individual 95% confidence intervals for each measurement.

The data sets supporting all results are included in a Microsoft Excel spreadsheet file, Additional file 1, containing charts and tables. High-resolution images for Figures 1 to 6 are included in a PDF file, Additional file 2, and a Microsoft PowerPoint file, Additional file 3.

### Folate immunoassays

#### Immunoassay serum folate

Serum folate (Figure 1C) was initially above the analyser limit of 54 nmol/L, a presumed consequence of an initial folate-replete diet. Serum folate responded without delay, after commencement of the folate-deficient diet on Day 0, rapidly falling to a stable level near the normal lower limit of 6.8 nmol/L in 147 days. There was no consistent fall below normal until after day 219, when it fell slowly but consistently to reach zero on day 525. Serum folate increased rapidly immediately after restoration of the normal folate-replete diet.

#### Immunoassay red-cell folate

The three clinical pathology laboratories reported very significantly different results for red-cell folate (Figure 1D). Results from Laboratory A initially vary randomly around 1000 nmol/L, with no significant response until day 343. There is then a decelerating fall, to below the normal lower limit of 360 nmol/L on day 420, reaching zero folate on day 574. Red-cell folate results from Laboratory B show an initial concentration of 2988 nmol/L, falling slowly but without significant delay and with initial severe instability, reaching a minimum of 879 nmol/L on day 469. Laboratory C shows an initial red-cell folate concentration of 2142 nmol/L, falling slowly but without significant delay, with slight instability, reaching a minimum of 535 nmol/L on day 525. All three laboratories reported a rapid increase in red-cell folate levels immediately following resumption of the folate-replete diet.

### Folate microbiological assay

The Food Science and Human Nutrition Department, University of Florida, provided a summary report of their results for red-cell folate and serum folate (unpublished; copy available from this author) for samples collected on day 574. The mean serum folate was 0.6 ± 0.09 nmol/L (n = 8), with an inter-assay CV of 5.4% and an intra-assay CV of 7.9%. The mean red-cell folate was 99 nmol/L, with an inter-assay CV of 12.5% and an intra-assay CV of 7.2%. The report noted: “*We have never analyzed samples with folate concentrations as low as these samples*”.

### Homocysteine

Serum total homocysteine was initially 9.9 μmol/L, varying around the recommended upper limit of 10.0 μmol/L, but with no significant change until day 323 (Figure 1B). The homocysteine concentration then increased exponentially, with some short-term variations, to reach a maximum of 49.3 μmol/L on day 553. Following the resumption of the folate-replete diet, the serum homocysteine concentration fell rapidly to 7.2 μmol/L.

### Haemoglobin

Haemoglobin was within the normal range at the commencement of the experiment (Figure 1A), remained without significant change until about day 469. It then fell linearly, to very significantly below the normal range, reaching a minimum near day 588, two days after resumption of the folate-replete diet. Haemoglobin then increased without further delay, returning to the initial level on day 772.

### Bone marrow examinations

The results of the first bone marrow examination were all normal except for mildly decreased iron stores, whereas those for the second clearly demonstrated megaloblastic anaemia. Cellularity of the aspirate was moderately increased, erythropoiesis showed moderately dyserythropoietic features, including megaloblastic changes and poor hemoglobinization. Some hypersegmented neutrophils were present but lymphocytes were normal. Plasma cells were normal and megakaryocytes were within normal limits; there was an occasional very large form with bizarre nuclear morphology. Iron stores were normal; an occasional ring sideroblast was present. Bone marrow surface markers were all normal. Trephine biopsy marrow was hypocellular in one half and normal in the other; trabeculae appeared normal. There was no evidence of malignant infiltration.

### Red-cell folate immunoassay performance

#### Siemens Advia Centaur

Laboratory A, using the Siemens Advia Centaur immunoassay system, consistently reported red-cell folate concentrations below the reference level defined by Bates and Lewis (2012) (Figure 2A), after day 413, following folate depletion. Laboratory A also consistently reported red-cell folate concentrations very significantly below their own minimum level (Figure 2B), after day 357. The red-cell folate concentration reported by the Siemens Advia Centaur was the only one that was consistent with the single-point result reported by the Food Science and Human Nutrition Department, University of Florida.

#### Roche Elecsys 2010

Laboratory B, using the Roche Elecsys 2010, never reported red-cell folate concentrations below the reference level defined by Bates and Lewis (2012) (Figure 2A). Laboratory B only once reported red-cell folate concentrations slightly below their own initial minimum level (Figure 2B). After their minimum normal level was increased very significantly, following the change in method towards the end of the depletion stage, the Roche Elecsys 2010 reported increasing red-cell folate concentrations that were initially below their new minimum level. Because of the change in method, the Roche Elecsys 2010 was not always able to report a numerical result for red-cell folate concentration. The Roche Elecsys 2010 result for the previous week was far higher than the single-point result reported by the Food Science and Human Nutrition Department, University of Florida.

#### Beckman UniCel DxI 800

Laboratory C, using the Beckman UniCel DxI 800, never reported red-cell folate concentrations below the reference level defined by Bates and Lewis (2012) (Figure 2A). Laboratory C consistently reported red-cell folate concentrations significantly below their own minimum level, following folate depletion (Figure 2B), after day 308. The red-cell folate concentration reported by the Beckman UniCel DxI 800 was far higher than the single-point result reported by the Food Science and Human Nutrition Department, University of Florida.

### Red-cell folate immunoassay error analysis

#### Comparison with lines of equality

The results from all three combinations of red-cell folate immunoassay methods were compared to the lines of equality and confidence intervals (Figure 3). Of the 85 results comparing the Siemens Advia Centaur and the Roche Elecsys 2010, none were within the 99.9% confidence interval (Figure 3A). Of the 91 results comparing the Siemens Advia Centaur and the Beckman UniCel DxI 800, 17 were within the 99.9% confidence interval (Figure 3B). Of the 83 results comparing the Beckman UniCel DxI 800 and the Roche Elecsys 2010, 16 were within the 99.9% confidence interval (Figure 3C).

The results comparing the Siemens Advia Centaur and the Beckman UniCel DxI 800 were weakly correlated, with a correlation coefficient of 0.75. The results comparing the Siemens Advia Centaur and the Beckman UniCel DxI 800 were also weakly correlated, with a correlation coefficient of 0.73. There was stronger correlation between the results from the Beckman UniCel DxI 800 and the Roche Elecsys 2010, with a correlation coefficient of 0.91.

#### Errors against mean

The errors, in standard deviations, between results from all three combinations of red-cell folate immunoassay methods were plotted against the mean (Figure 4). Of the 85 results comparing the Siemens Advia Centaur and the Roche Elecsys 2010, two were within the 99.9% confidence interval (Figure 4A). Of the 91 results comparing the Siemens Advia Centaur and the Beckman UniCel DxI 800, 22 were within the 99.9% confidence interval (Figure 4B). Of the 83 results comparing the Beckman UniCel DxI 800 and the Roche Elecsys 2010, 37 were within the 99.9% confidence interval (Figure 4C).

#### Errors over time

The errors, in standard deviations, between results from all three combinations of red-cell folate immunoassay methods were plotted against time (Figure 5). The maximum difference between the Siemens Advia Centaur and the Roche Elecsys 2010 was 16 standard deviations, on day 504 (Figure 5A). The maximum difference between the Siemens Advia Centaur and the Beckman UniCel DxI 800 was 17 standard deviations, on days 575 and 584 (Figure 5B). The maximum difference between the Roche Elecsys 2010 and the Beckman UniCel DxI 800 was nine standard deviations, on day 22 (Figure 5C).

#### Variation over time, with error limits

The results from all three red-cell folate immunoassay methods, with the 95% confidence intervals, were plotted against time (Figure 6). None of the results from Laboratory A were initially within the 95% confidence interval of the results from either of the other laboratories. The results then tend to converge, with 10 out of 91 results from Laboratories A and C being within the 95% confidence interval, but none of the 85 results from Laboratories A and B being within the 95% confidence interval. The results rapidly diverge after day 343, with no results from Laboratory A, and either of the other two laboratories, being within the 95% confidence interval after day 378.

## Discussion

The fall in haemoglobin concentration, to a level well below that defined by World Health Organization (2011) for moderate anaemia, and the finding of megaloblastic changes and poor haemoglobinisation in the second bone marrow examination, confirm the development of megaloblastic anaemia. The very low serum and red-cell folate concentrations, reported by the University of Florida, confirm that the subject was severely depleted of folate. The rise in serum total homocysteine concentration, to well above the maximum normal level, confirms that severe cellular folate deficiency had developed.

Only one of the three laboratories reported red-cell folate results that were consistent with the evidence for folate deficiency, as defined by the reference level of Bates and Lewis (2012). Laboratory A, using the Siemens Advia Centaur immunoassay system, consistently reported red-cell folate concentrations very significantly below the reference level.

Two of the three laboratories reported red-cell folate results that were consistent with the evidence for folate deficiency, as defined by their own minimum levels. Laboratory A, using the Siemens Advia Centaur, and Laboratory C, using the Beckman UniCel DxI 800, consistently reported red-cell folate concentrations significantly below their own minimum levels, following folate depletion. Laboratory B, using the Roche Elecsys 2010, reported red-cell folate concentrations below their own minimum level only after that minimum level was significantly increased by the laboratory when megaloblastic anaemia had developed.

The very significant differences, between the results reported by the three laboratories, varied with time and folate concentration. The differences were greatest, up to 17 standard deviations, between the Siemens Advia Centaur and each of the Roche Elecsys 2010 and the Beckman UniCel DxI 800. Less than a quarter of the results for the Siemens Advia Centaur, and those from each of the other two systems, were within a 99.9% confidence interval. Less than half of the results from the Roche Elecsys 2010 and the Beckman UniCel DxI 800 were within a 99.9% confidence interval.

### Potential explanations for immunoassay differences

#### Immunoassay interference

A potential explanation for the very significant differences in results between the three immunoassay methods is immunoassay interference (Selby 1999; Kricka 2000; Tate and Ward 2004). Antibodies in the subject’s blood can compete with antibodies used in the immunoassay kits. The type of antibody used, and the extent of warnings provided, varies between the immunoassay kit manufacturers (Siemens 2004; Roche Diagnostics 2011; Beckman Coulter 2008). The absence of any significant difference in results between the Siemens Advia Centaur and the Roche Elecsys 2010, for serum folate, suggests that any interference cannot be caused by antibodies in the serum.

#### Sample preparation

The most likely explanation for the very significant differences in results, between the three immunoassay methods, is difference in sample preparation (Gilois et al. 1990; Wright et al. 1998, 2000; Netteland and Bakke 1977; Omer 1969). All three immunoassay systems use different methods of sample preparation (Siemens 2004; Roche Diagnostics 2011; Beckman Coulter 2008). There is a high correlation between red-cell folate results from the Roche Elecsys 2010 and the Beckman UniCel DxI 800, but not between those systems and the Siemens Advia Centaur. This, together with the similarity in results between the Siemens Advia Centaur and the Roche Elecsys 2010 for serum folate, suggest differences in sample preparation as the most likely cause for the very significantly different results for red-cell folate.

### Unanswered questions

#### Qualitative response to folate depletion

Why did the Roche Elecsys 2010 and the Beckman UniCel DxI 800 report significantly falling red-cell folate concentrations in the first 330 days, whereas the Siemens Advia Centaur reported no change in that period (Figures 1D, 2 and 6)? This question needs to be considered in the context of the observation that the Siemens Advia Centaur was the only immunoassay method to report results that were consistent with the microbiological assay; it was also the only immunoassay to report folate deficiency, as defined by the reference level of Bates and Lewis (2012).

#### Change in method for the Roche Elecsys 2010

Why was there a change in sensitivity of the Roche Elecsys 2010, towards the end of the folate-depletion stage? This question needs to be considered in the context of the fact that the change was in the instrument software only, with the hardware and reagents unchanged. The effect of the change was to very significantly increase the minimum reportable concentration of red-cell folate, preventing meaningful reporting of some of the later results.

#### Change in minimum of reference range for the Roche Elecsys 2010

Why was there a very significant increase in the minimum of the reference range for the Roche Elecsys 2010, towards the end of the folate-depletion stage (Figure 1D)? Without this change, this immunoassay would not have reported any folate deficiency, as defined by the laboratory’s own reference level, even when megaloblastic anaemia was most severe.

### Limitations of the experiment

#### Number of subjects

For ethical reasons, a longitudinal experiment designed to produce megaloblastic anaemia can only be performed by means of self-experimentation on a single subject. It is therefore not possible to produce comparative longitudinal red-cell folate immunoassay data for a large group of subjects. The results for this single subject do provide unique longitudinal data that is consistent with the two published comparison studies for the three immunoassays used in this experiment (Callen 2010; Owen and Roberts 2003).

#### Number of laboratories

In an ideal world, to eliminate individual laboratory error, it would be desirable to have samples tested by more than one laboratory utilizing each immunoassay method. This was not practical because of logistical problems, including the need to draw excessive volumes of blood from the subject on each sampling day. With modern automated immunoassay systems, with use of pre-packaged reagent kits, the likelihood of significant individual laboratory error has been reduced. Taking into account the strict quality control procedures required, and the very large number of sampling days over such a long time span, it is unlikely that the differences can be explained by individual laboratory error.

## Conclusions

Comparative longitudinal data from clinical pathology laboratories, produced during experimental folate deficiency, have exposed very significant differences in results between commercial red-cell folate immunoassays. One immunoassay, the Roche Elecsys 2010, failed to detect overt megaloblastic anaemia of severe folate deficiency.

### Primary data

All primary data, as scanned PDF copies of pathology reports, are available from the author.

## Electronic supplementary material

Additional file 1: **Severe Experimental Folate Deficiency Part B - for Figures 1 to 6, tables and charts.** (XLSX 988 KB)

Additional file 2: **Figures 1 to 6, High-resolution images.** (PDF 142 KB)

Additional file 3: **Figures 1 to 6, High-resolution slides.** (PPTX 733 KB)
