# Supplementary material for: Severe experimental folate deficiency in a human subject - a longitudinal investigation of red-cell folate immunoassay errors as megaloblastic anaemia develops
Source: Springerplus. 2014 Sep 23;3:441. doi: 10.1186/2193-1801-3-441 (PMC4190184; doi:10.1186/2193-1801-3-441)
Supplement: Supplementary file 2 — Additional file 2: Figures 1 to 6, High-resolution images. (PDF 142 KB) [file 40064_2014_1257_MOESM2_ESM.pdf]

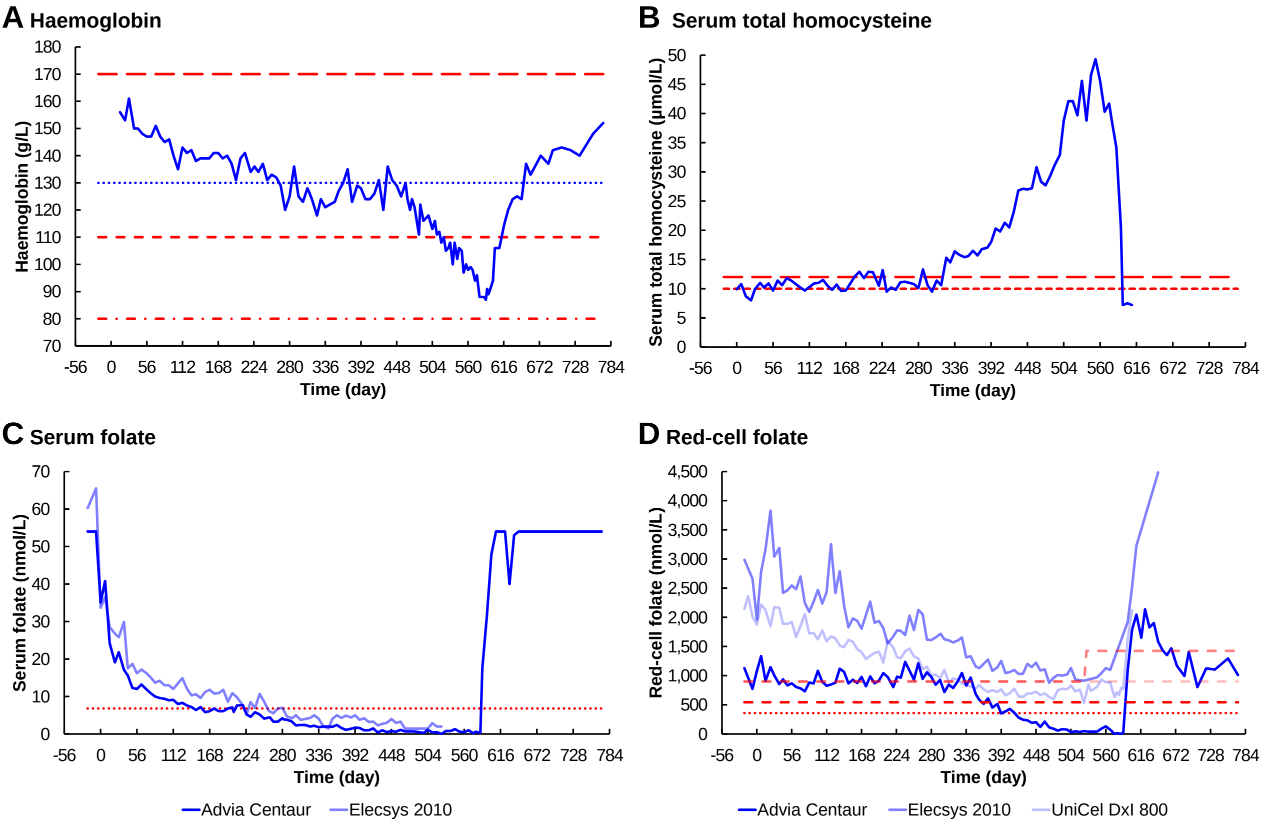
A Haemoglobin
Haemoglobin (g/L)
180
170
160
150
140
130
120
110
100
90
80
70
-56 0 56 112 168 224 280 336 392 448 504 560 616 672 728 784
Time (day)
B Serum total homocysteine
Serum total homocysteine (µmol/L)
50
45
40
35
30
25
20
15
10
5
0
-56 0 56 112 168 224 280 336 392 448 504 560 616 672 728 784
Time (day)
C Serum folate
Serum folate (nmol/L)
70
60
50
40
30
20
10
0
-56 0 56 112 168 224 280 336 392 448 504 560 616 672 728 784
Time (day)
Advia Centaur
Elecsys 2010
D Red-cell folate
Red-cell folate (nmol/L)
4,500
4,000
3,500
3,000
2,500
2,000
1,500
1,000
500
0
-56 0 56 112 168 224 280 336 392 448 504 560 616 672 728 784
Time (day)
Advia Centaur
Elecsys 2010
UniCel DxI 800

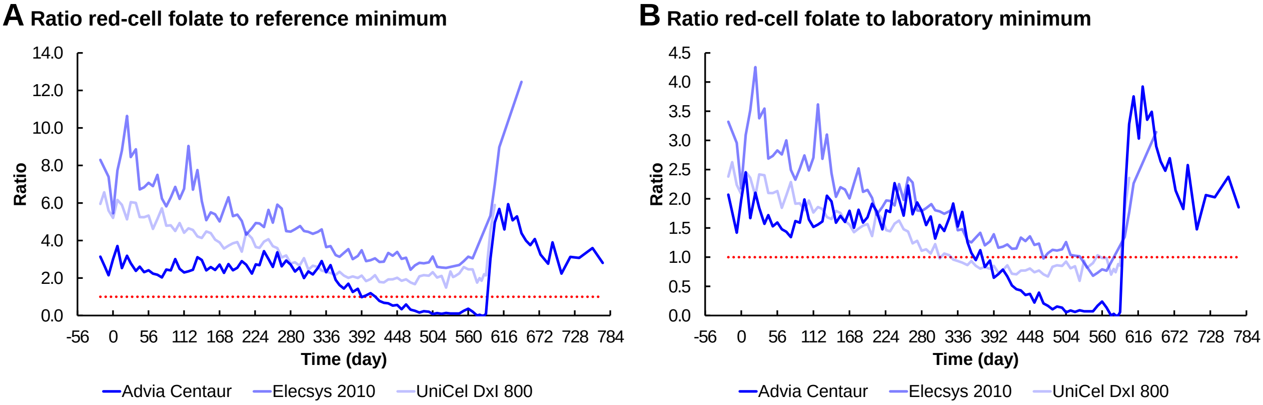
A Ratio red-cell folate to reference minimum
14.0
12.0
10.0
8.0
6.0
4.0
2.0
0.0
Ratio
-56
0
56
112
168
224
280
336
392
448
504
560
616
672
728
784
Time (day)
Advia Centaur
Elecsys 2010
UniCel DxI 800
B Ratio red-cell folate to laboratory minimum
4.5
4.0
3.5
3.0
2.5
2.0
1.5
1.0
0.5
0.0
Ratio
-56
0
56
112
168
224
280
336
392
448
504
560
616
672
728
784
Time (day)
Advia Centaur
Elecsys 2010
UniCel DxI 800

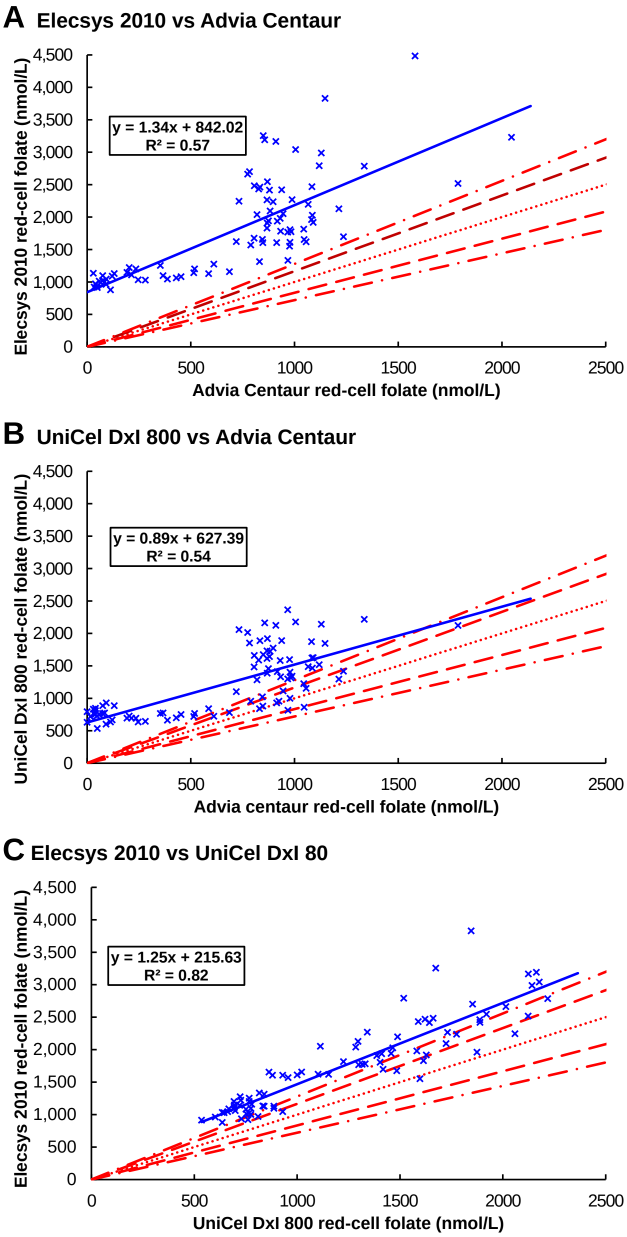
A Elecsys 2010 vs Advia Centaur
Elecsys 2010 red-cell folate (nmol/L)
Advia Centaur red-cell folate (nmol/L)
y = 1.34x + 842.02
R² = 0.57
0
500
1,000
1,500
2,000
2,500
3,000
3,500
4,000
4,500
0
500
1000
1500
2000
2500
B UniCel DxI 800 vs Advia Centaur
UniCel DxI 800 red-cell folate (nmol/L)
Advia centaur red-cell folate (nmol/L)
y = 0.89x + 627.39
R² = 0.54
C Elecsys 2010 vs UniCel DxI 80
Elecsys 2010 red-cell folate (nmol/L)
UniCel DxI 800 red-cell folate (nmol/L)
y = 1.25x + 215.63
R² = 0.82

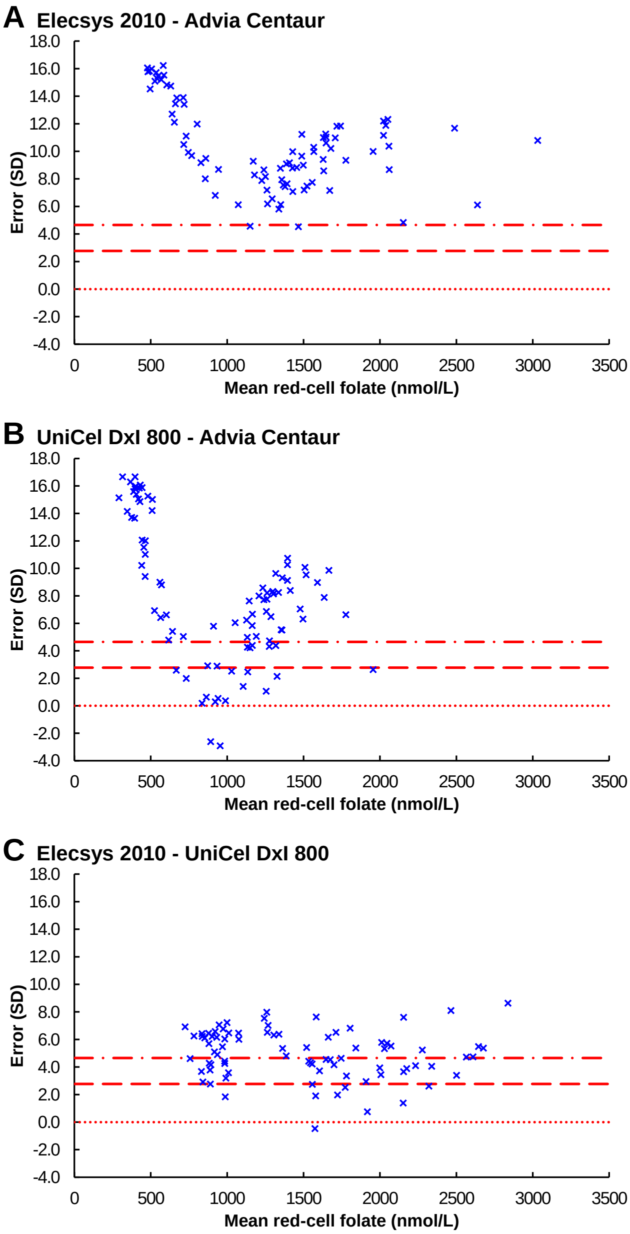
A
Elecsys 2010 - Advia Centaur
Error (SD)
18.0
16.0
14.0
12.0
10.0
8.0
6.0
4.0
2.0
0.0
-2.0
-4.0
0
500
1000
1500
2000
2500
3000
3500
Mean red-cell folate (nmol/L)
B
UniCel DxI 800 - Advia Centaur
Error (SD)
Mean red-cell folate (nmol/L)
C
Elecsys 2010 - UniCel DxI 800
Error (SD)
Mean red-cell folate (nmol/L)

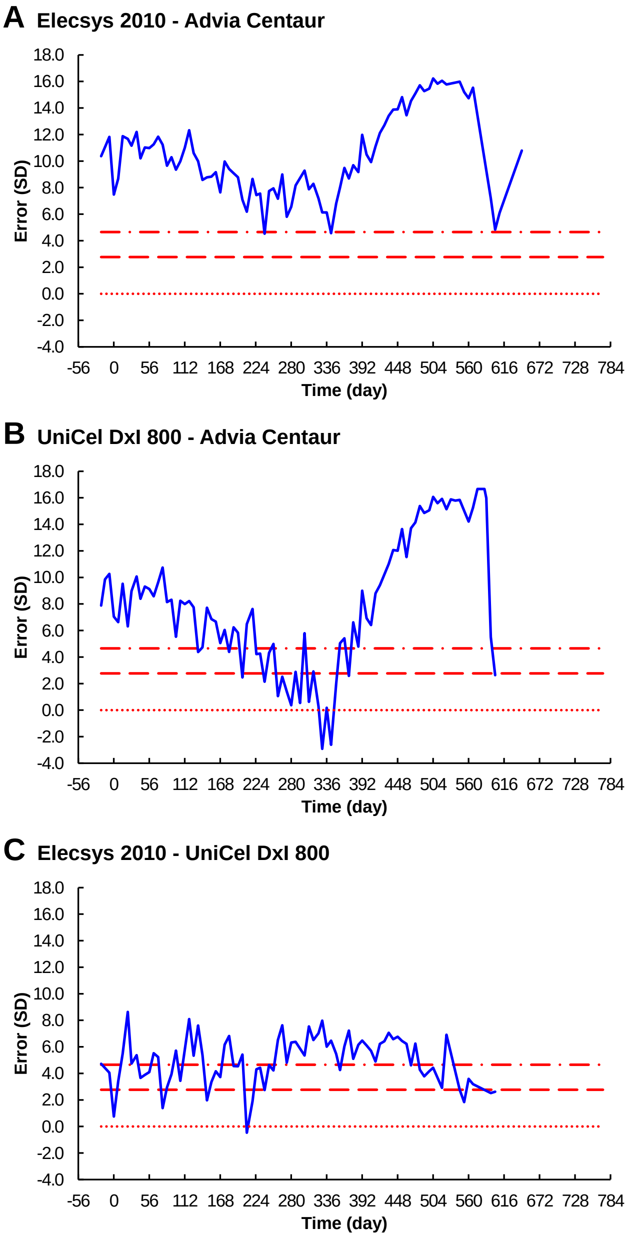
A Elecsys 2010 - Advia Centaur
B UniCel DxI 800 - Advia Centaur
C Elecsys 2010 - UniCel DxI 800
Error (SD)
18.0
16.0
14.0
12.0
10.0
8.0
6.0
4.0
2.0
0.0
-2.0
-4.0
-56
0
56
112
168
224
280
336
392
448
504
560
616
672
728
784
Time (day)

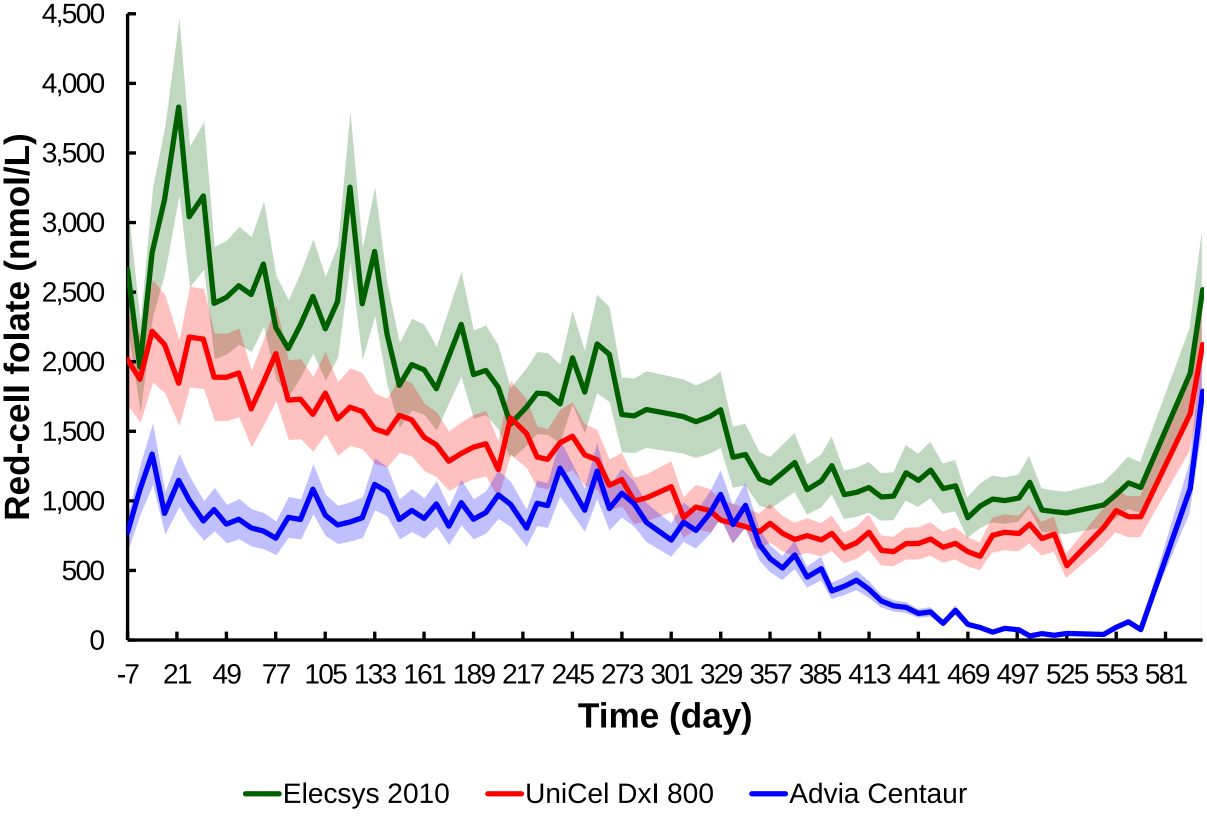

Red-cell folate (nmol/L)
4,500
4,000
3,500
3,000
2,500
2,000
1,500
1,000
500
0
-7
21
49
77
105
133
161
189
217
245
273
301
329
357
385
413
441
469
497
525
553
581
Time (day)
Elecsys 2010
UniCel DxI 800
Advia Centaur
