# Supplementary figures and images for: Severe experimental folate deficiency in a human subject - a longitudinal investigation of red-cell folate immunoassay errors as megaloblastic anaemia develops
Source: Springerplus. 2014 Sep 23;3:441. doi: 10.1186/2193-1801-3-441 (PMC4190184; doi:10.1186/2193-1801-3-441)

## Slide 1
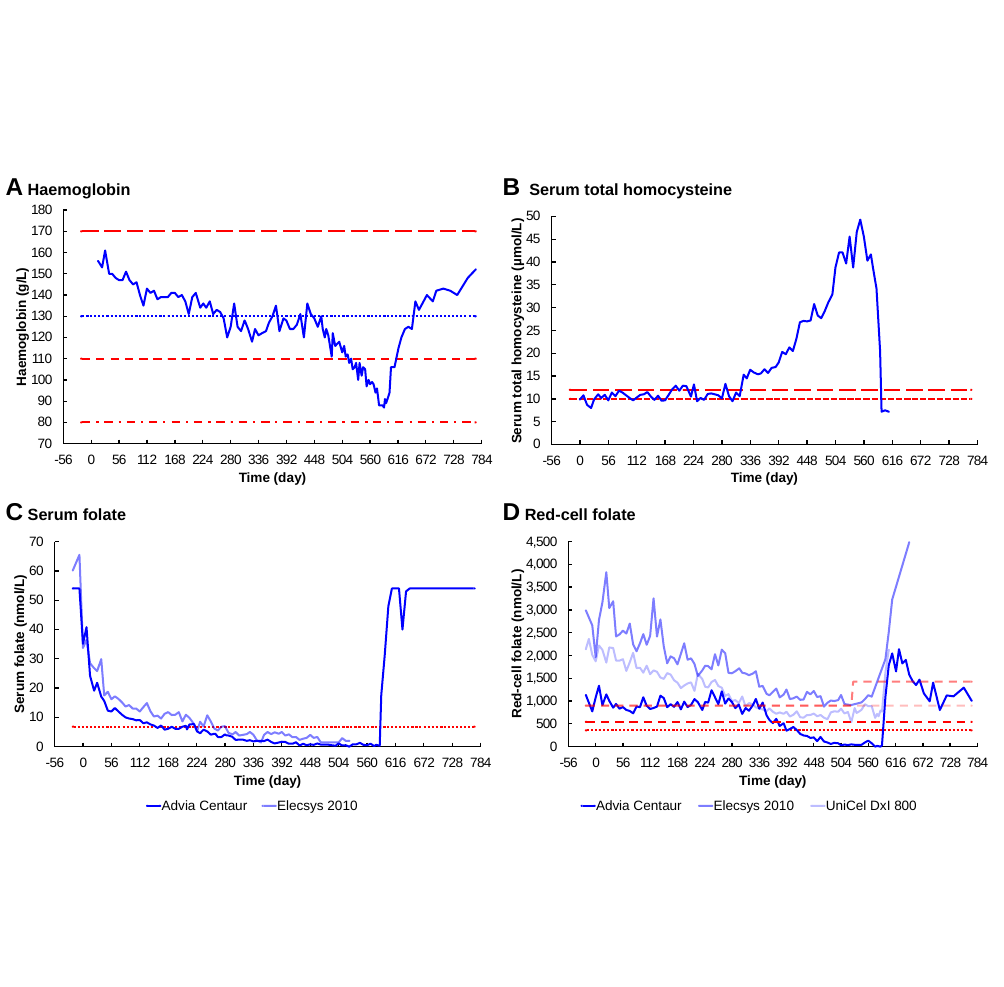

## Slide 2
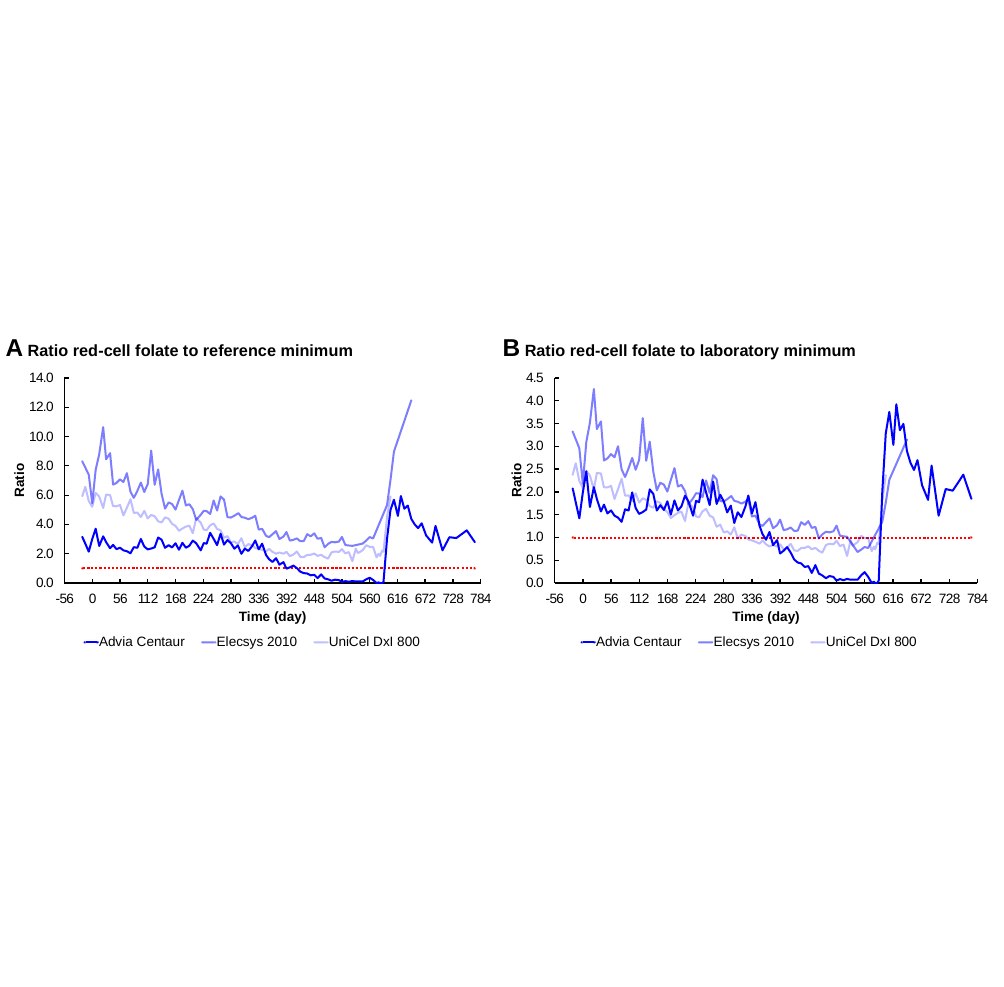

## Slide 3
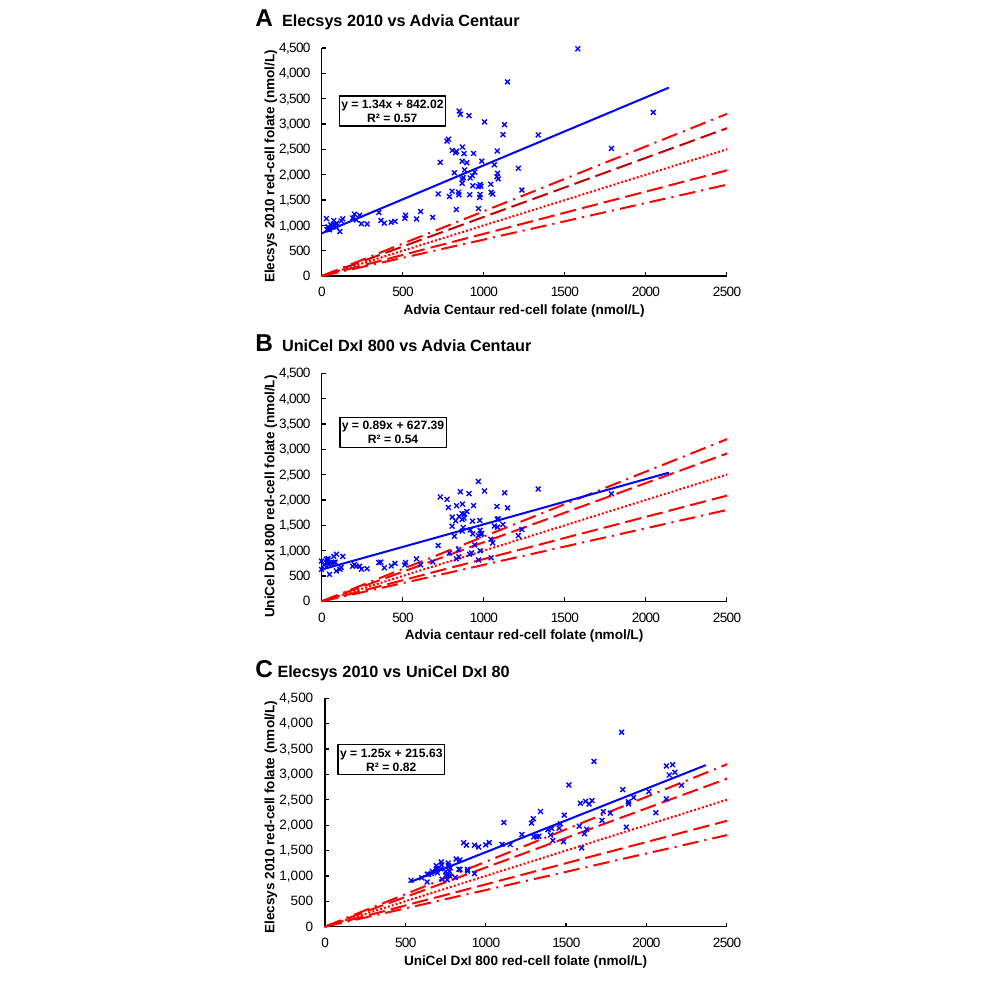

## Slide 4
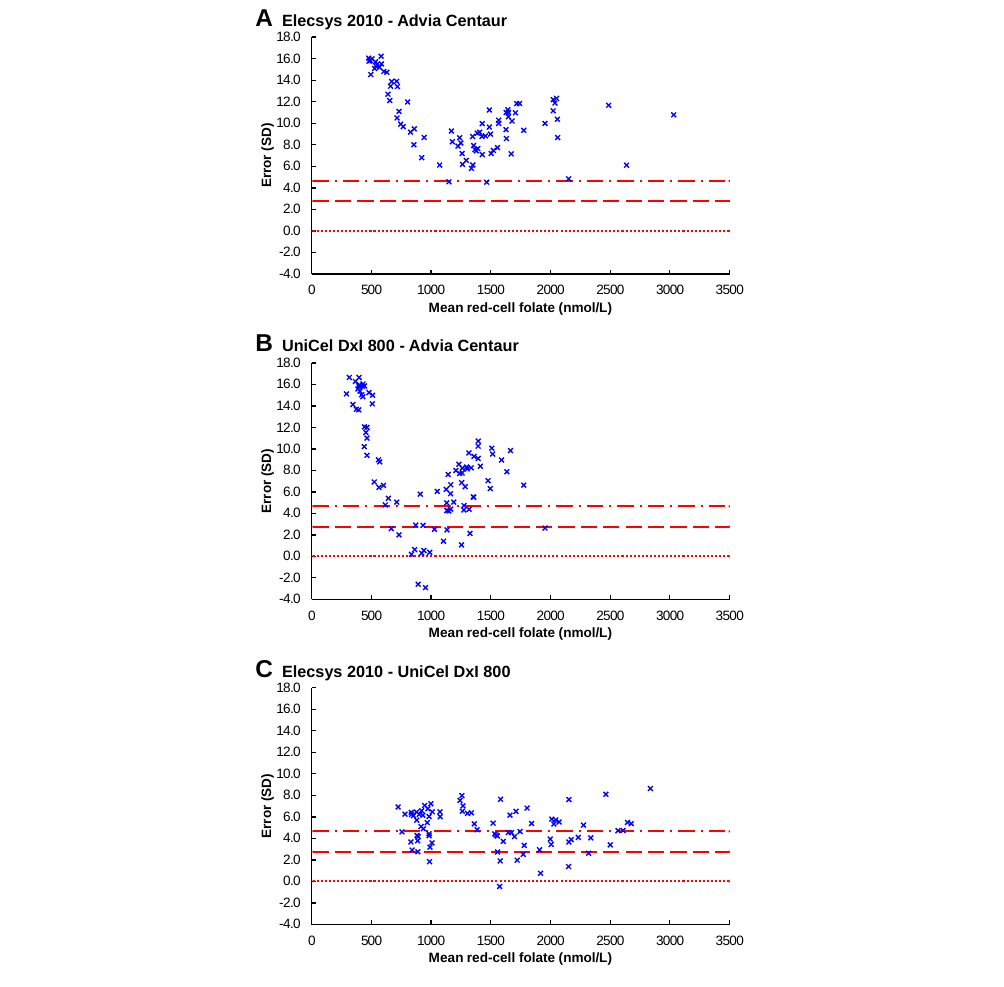

## Slide 5
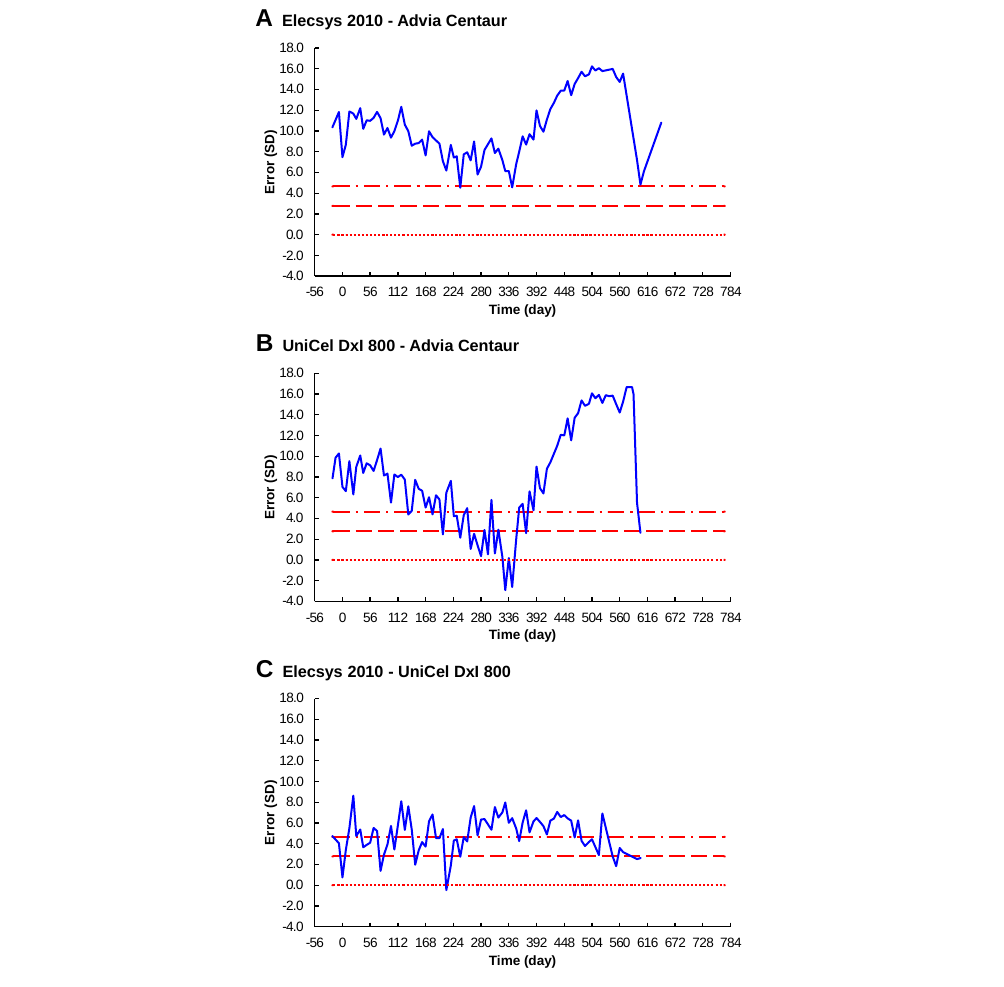

## Slide 6
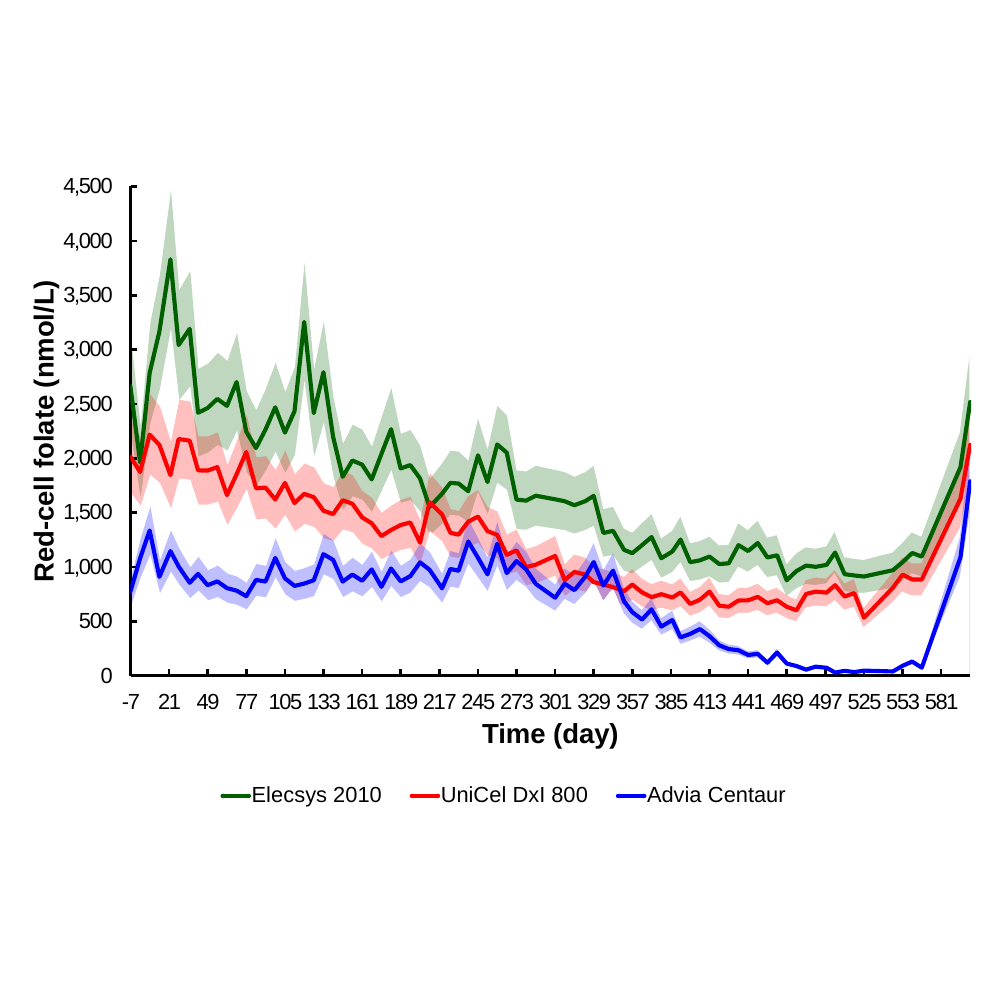

Supplement: Supplementary file 3 — Additional file 3: Figures 1 to 6, High-resolution slides. (PPTX 733 KB) [file 40064_2014_1257_MOESM3_ESM.pptx]
